# Treatment of Advanced Metastatic Prostate Cancer Using Molecular-Targeted Therapy: Radioligand Lutetium-177 Prostate-Specific Membrane Antigen

**DOI:** 10.7759/cureus.37778

**Published:** 2023-04-18

**Authors:** Rajni Rathore, Shahid B Rangrej, Ian Kieme, Victoria Carvalho, Katie King, Yacoubou Amadou, John McKinley, Audrey Masawi

**Affiliations:** 1 Pharmacology and Therapeutics, Saint James School of Medicine, Arnos Vale, VCT; 2 Anatomy/Research, Saint James School of Medicine, Arnos Vale, VCT; 3 Medical School, Saint James School of Medicine, Arnos Vale, VCT

**Keywords:** prostate cancer, molecular-targeted therapy, [177lu]lu-psma, radioligand [177lu]lu-psma, advanced metastatic prostate cancer

## Abstract

This study investigates the predicting factors of the biochemical response and survival of patients with advanced metastatic prostate cancer who underwent therapy with radioligand lutetium-177 (^177^Lu)-prostate-specific membrane antigen (PSMA), often referred to as [^177^Lu]Lu-PSMA. This study is a review of the previous literature. This study included articles published in the last 10 years in the English language. According to the literature review, treatment with [^177^Lu]Lu-PSMA has a positive impact on prostate-specific antigen (PSA) within the first cycle and a negative impact on lymph node metastasis. There is a plausible positive impact on PSA after multiple cycles and performance status and a negative impact on visceral metastasis. In conclusion, the reviews show that treatment with [^177^Lu]Lu-PSMA in patients with castration-resistant prostate cancer is beneficial in reducing PSA and metastasis.

## Introduction and background

Prostate cancer is the second most common type of cancer in American men. According to the American Cancer Society, annually, 205,853 males are diagnosed with prostate cancer with a mortality rate of 33,330 per year [1]. Localized tumors that are detected early are significantly curative whereas progressing and metastasized cancers have high mortality [2]. According to the Centers for Disease Control and Prevention, the 10-year prostate cancer relative survival for localized, regional, unknown, and distant cancers was 100%, 96.1%, 78.1%, and 18.5%, respectively for the 2001-2016 period [3]. Given the considerable disproportional mortality rate between early-stage and late-stage cancer, it is imperative that we evaluate new treatments to bridge the gap.

Treatment options for metastasized prostate cancer are based on patient age, the cancer growth rate, treatment benefits over drawbacks, and staging, further limiting the available therapeutic options [4]. There is an additional reduction in treatment options because some cancers become resistant to androgen deprivation therapy (ADT) with luteinizing hormone analogs such as leuprolide which is often given with or without an androgen receptor antagonist such as goserelin [4]. Metastasized androgen-resistant cancer is currently treated with chemotherapy drugs such as docetaxel, mitoxantrone, and cabazitaxel; hormone manipulation therapy with enzalutamide abiraterone, apalutamide, and olaparib; radiotherapy using radon-223 if bone metastasis is present; external-beam radiotherapy (EBRT) if cancer is not extensive in volume; and immunotherapy with sipuleucel-T if asymptomatic or mild symptoms with pembrolizumab [5].

A constant challenge to treating prostate cancer is acquired resistance to hormone therapy and treatment with docetaxel [6]. Many patients with advanced cancer become unresponsive to ADTs, referred to as having metastatic castration-resistant prostate cancer (mCRPC) which has shown positive treatment response with lutetium-177 (^177^Lu)-prostate-specific membrane antigen (PSMA)-617 [6]. It is a radioligand therapy that uses a β-emitting radioisotope lutetium-177 which is added to the ligand PSMA-617. This targets PSMA, a transmembrane glycoprotein that is expressed at high levels in prostate cancer. The ability to target specific cancer cells mitigates the systemic tissue damage that β radiation would cause [7].

An increase or decrease in prostate-specific antigen (PSA) levels from a baseline value is used to determine treatment success or failure [4]. Several studies included in this review reported notable decreases in PSA levels [6,8,9,10]. Further, studies that examined the efficacy and safety of ^177^Lu-PSMA-617 proved it to be promising [6,10,11]. Although ^177^Lu-PSMA-617 has shown notable results in the treatment of mCRPC, there is no consensus on its dose schedule. At present, there is limited knowledge regarding the number of cycles required to attain a PSA level ≥50.

This review aimed to investigate the factors predicting biochemical response and survival benefits following radioligand therapy with [^177^Lu]Lu-PSMA in advanced metastatic prostate cancer.

## Review

Design and methods

This study aims to perform a systematic literature search using Saint James School of Medicine library resources. Additional databases utilized included PubMed, EBSCO, Medline, and Cochrane Library. Search terms were “177Lu-PSMA,” “radioligand” “response to therapy,” “prognosis,” and “predict.” In this study, we included relevant articles published within the last 10 years in the English language only. Chosen articles discussed one or more factors which may be prognostic or predictive of the response to [^177^Lu]Lu-PSMA, that is, PSA response and survival parameters. Studies published outside the 10-year timeframe and not in the English language were excluded.

Results

Table 1 depicts the factors used to assess PSA levels and their impact.

**Table 1:** Factors used to assess PSA levels according to various filters. PSA: prostate-specific antigen

Assessment	Impact	Strength
Any PSA decline after the first cycle	Positive	Confirmed
Gleason score	Negative	Confirmed
Any PSA decline after a few cycles	Positive	Plausible
>50% PSA decline after a few cycles	Positive	Plausible
Better performance status	Positive	Plausible
Lymph node metastasis	Positive	Plausible
Visceral metastasis	Negative	Plausible

Confirmed Positive Impact: Any PSA Decline After the First Cycle

A study distributed the European Organization for the Research and Treatment of Cancer Quality of Life Questionnaire to 30 patients [12], with ages ranging from 50 to 87, who had undergone ^177^Lu-PSMA-617 therapy from 2014 to 2016. The questionnaire was used to assess their quality of life two months before starting the therapy and two months after participating in the study. The study gathered and compared data based on changes in the following three categories: (A) global health status, (B) disease-related symptoms, and (C) the effects of PSA values. The majority of patients participated in three treatment cycles (n = 12), a minimum of two (n = 6), and a maximum of eight (n = 1) cycles were administered. After the first cycle, the response to the therapy was 73% (n = 22). A two-month follow-up was performed after the therapy to assess quality of life, which revealed an increase in global health status (p = 0.025) and emotional functioning (p = 0.010) and a decrease in pain levels (p = 0.033). As p-values were less than 0.05, there was statistical significance and a strong correlation between PSA treatment and improvement in quality of life per cycle.

In a study published in The Lancet [13], an experiment in Australia was used to compare the effects of cabazitaxel with that of [^177^Lu]Lu-PSMA-617 in patients with mCRPC. The study was performed as an unblinded, phase 2 trial at 11 locations. The patients who were recruited were men confirmed as having mCRPC and cabazitaxel as their next most appropriate treatment based on protocol. Before being allowed to participate, the volunteers were required to test for adequate renal, liver, and hematological function as that could impair the results of the study. They were then randomly assigned on a 1:1 ratio and were administered either [^177^Lu]Lu-PSMA-617 (6.0-8.5 GBq intravenously every six weeks for up to six cycles) or cabazitaxel (20 mg/m^2^ intravenously every three weeks for up to 10 cycles). From February 2018 to September 2019, 291 men were screened. A vast majority (99%) of the volunteers randomly assigned to the [^177^Lu]Lu-PSMA-617 treatment responded versus those assigned to cabazitaxel (85%). There were fewer grade 3-4 adverse effects in those who received [^177^Lu]Lu-PSMA-617 compared to cabazitaxel, with the findings reporting 33% and 53%, respectively. Therefore, it can be said that [^177^Lu] Lu-PSMA-617 is an effective alternative therapy to cabazitaxel for the treatment of mCRPC.

In a study published by the Journal of Nuclear Medicine [14], 56 volunteers (with a median age of 72 years) underwent 125 cycles of ^177^Lu-PSMA. Specifically, they were given one cycle for 16 patients, two cycles for 15 patients, three for 17 patients, four for six patients, and five for two patients. The range of ^177^Lu-PSMA per cycle was 3.6-8.7 GBq, with the median dose administered being 5.76 GBq. The PSA response was documented monthly, with a follow-up for a median of 15 months for 25 patients who underwent additional therapy cycles. Those who were followed up underwent two cycles for nine patients, three for seven patients, and four for nine patients.

The results demonstrated that 45/56 patients (80.4%) experienced a decrease in PSA levels. Before beginning the trial, the median PSA levels were 43.2 ng/mL (range = 0.05-2,848 ng/mL), which after treatment reduced to 23.8 ng/mL (range = 0.01-2,227 ng/mL). A decline of greater than 80% was seen in 13 (23.2%) patients, a decline of greater than 50% was seen in 33 (58.9) patients, and a decline by greater than 30% was seen in 33 (66.1%) patients. However, some patients demonstrated an increase in PSA levels as well as an increase in lymph node metastasis. In addition, there were no strict criteria for the selection of the volunteers in contrast to previous sources, and the patients were a heterogenous group [14].

An additional study by the Journal of Nuclear Medicine had 82 volunteers (age range = 43-87 years) who had confirmed mCRPC. The patients were given a single dose of [^177^Lu]Lu-PSMA-617, with a mean dose of 5.9 ± 0.5 GBq. Data were gathered when the patients were evaluated at baseline and eight weeks after the administration of therapy. Screening for patients was done by the prerequisite of disease progression despite first and second-line chemotherapeutic options (docetaxel and cabazitaxel, respectively). A positive aspect of this experiment was that to reduce the uptake of [^177^Lu] Lu-PSMA-617 by the salivary glands, it was cooled with cold packs 30 minutes before the injection [15]. Not every patient survived the treatment with six patients dying in the process. Two patients had also dropped out, leaving the data of only 74 patients available. Of the 74 patients, 47 (64%) patients showed a decline in PSA levels, with 23/47 showing a decline of more than 50%. However, 17 of the 74 (23%) patients were classified as experiencing disease progression as their PSA levels increased by at least 25%. The study also reported the first case series of 10 patients who had received a single dose of ^177^Lu-PSMA-617. The study findings demonstrated a low toxicity profile and a decrease in PSA levels by more than 50% and 30% or more in 50% and 70% of the patients [15].

Lastly, another study involved 24 patients with ages ranging from 64-82 years, with a median age of 75 years. At baseline, the median PSA level was reported to be 522 ng/mL. One patient died during the process 10 weeks after the administration of the first cycle. He was noted to have had multiple liver metastases and ECOG 3. A response evaluation was given eight weeks after the first cycle, where 19/24 (79.1%) patients experienced a PSA decline. Of the 19 patients, 13 experienced a decline of more than 30%, and 10 experienced a decline of more than 50% [16].

Confirmed Negative Impact: Gleason Score

A retrospective study examined 40 mCRPC patients who had undergone ^177^Lu-PSMA-617 PSMA radioligand therapy treatment. Out of 40 cases, 21 were responders and 19 were non-responders. 18-Fludeoxyglucose (FDG) positron emission tomography (PET) findings uptake was noted in 35/40 patients (87.5%). Patients with negative serum PSA doubling time demonstrated superior one-year progression-free survival compared to those with positive serum PSA doubling time (52.5 vs. 47.5%) (p = 0.029). Patients receiving greater than two cycles of ^177^Lu-PSMA-617 PSMA-targeted radioligand therapy demonstrated a higher negative PSA doubling time compared to those receiving two cycles (p = 0.03). FDG uptake was observed with aggressive disease biology coupled with increasing Gleason score and poorer 12-month progression-free survival. Negative PSA doubling time following therapy demonstrated longer progression-free survival. Patients with Gleason scores of 8 and above showed higher FDG uptake; hence, a high FDG uptake with a high Gleason score is indicative of aggressive disease biology [8].

A study of 71 patients with castration-resistant metastatic prostate cancer who had gallium (Ga)-PSMA-11 PET/CT scan and then were treated with ^177^Lu-PSMA-617 radioligand therapy over a three-year period. These patients had unsuccessful treatment with other therapies. All lesions were analyzed by two different readers and isocontour volume was created. This allowed the whole-body total lesion and whole-body PSMA-TV to be calculated [17]. Localization of all lesions was also noted which allowed standardized uptake value to be calculated and used for the analysis of long-term outcomes. Treatment was given on a 6-8-week cycle and response was assessed during cycle 3 by PSA levels. If the PSA decreased by greater than 50%, it was considered a response to treatment. The PSA response was recorded in a waterfall plot. The Gleason score of this study was 8 in both responders and non-responders. This study did not find any association between PSA decline and the Gleason score [17]. One-way analysis of variance was used to determine if there was a difference between PSMA-TV, TL-PSMA, or PSMA expression and Gleason score or tumor localization.

Plausible Positive Impact: Lymph Node Metastases

In a study by Siegel et al., prostate cancer was the most common cancer in men and the second leading cause of cancer death in men in the United States [18]. Lymph node metastasis is the most common cause of death from prostate cancer. The number of positive lymph nodes is an important predictor of recurrence and survival. In another study by Abufaraj et al., it was revealed that in a regional analysis, the sensitivity of ^68^Ga-PSMA-11 PET/MRI was 72-100% [19]. This is important because it means that this tracer can be used to accurately stage lymph node metastases in prostate cancer patients. ^68^Ga-PSMA PET is a more accurate way to stage prostate cancer and guide treatment decisions, which can potentially save lives.

Lymph node metastasis is the most common type of cancer spread. It occurs when cancer cells from the primary tumor travel through the lymphatic system to nearby lymph nodes. Lymph node metastasis is a serious condition that can lead to significant morbidity and mortality. However, there is evidence that lymph node metastasis is not always an indicator of a poor prognosis. In some cases, patients with lymph node metastasis have been shown to have a better prognosis than those without lymph node metastasis. This is thought to be because the presence of cancer cells in the lymph nodes can help the immune system better target and destroy the cancer cells. Factors predicting biochemical response and/or survival in patients with metastatic disease are largely unknown. However, some studies have suggested that the presence of lymph node metastasis may be a positive prognostic factor in patients with metastatic disease. A study by ManafiFarid et al., that reviewed the literature on patients with mCRPC who underwent radioligand therapy with [^177^Lu]Lu-PSMA found that the presence of lymph node metastasis was associated with a higher rate of biochemical response and longer progression-free survival [20]. These findings suggest that lymph node metastasis may be a positive prognostic factor in patients with mCRPC. In addition, the authors found that patients with a higher number of lymph nodes involved had a better response to radioligand therapy. A meta-analysis evaluating the site of metastasis on the overall survival of men with mCRPC suggested that the more extensive the lymph node metastasis, the more likely it is to be a positive prognostic factor. Therefore, the presence of lymph node metastasis should not be automatically considered a negative prognostic factor in patients with metastatic disease [21].

Nuclear medicine in prostate cancer is a well-established field that uses various radiopharmaceuticals to visualize and target prostate cancer cells. Nuclear medicine imaging techniques such as PET and single-photon emission computed tomography can be used to detect and stage prostate cancer. In addition, nuclear medicine therapies such as radionuclide therapy and radioimmunotherapy can be used to treat prostate cancer. PET is a nuclear medicine imaging. It is uncertain whether early detection and eradication of low-volume nodal recurrences affect the disease course in patients who experience biochemical recurrence within five years. In a study by De Bruycker et al., after metastasis-directed therapy for PET-detected recurrences, two prospective phase 2 trials showed encouraging outcomes, with good local control and low metastasis rates and castration-resistant disease [22]. However, most patients experience a biochemical recurrence within one year, which is followed by a clinical recurrence. When using new imaging to target the brain, these findings highlight the drawbacks of a lesion-directed strategy. In other words, the authors found that patients who received treatment for their metastatic disease had a better outcome if they were treated early before the disease progressed. This suggests that early detection and treatment of metastatic disease is important for patients with prostate cancer. The results of De Bruycker et al. support the possible benefit of more comprehensive approaches such as elective nodal radiotherapy and super-extended salvage lymph node dissection in the management of patients with biochemical recurrence [22]. This shows that there may be a benefit to treating the lymph nodes even when the disease has not yet spread to them.

A study by Luiting et al. revealed that in primary prostate cancer, an extended pelvic node dissection (PLND) is the gold standard for lymph node staging, but lymph node metastasis can be omitted during lengthy PLND due to the technical difficulties in removing all lymph nodes [23]. In recent years, new imaging modalities such as ultrasound, CT, and MRI have been used to improve the detection of nodal metastasis and guide the extent of dissection. However, it is unclear whether these new modalities can improve the staging and prognostication of prostate cancer. The sensitivity of ^68^Ga-PSMA PET is influenced by the number of nodes removed when compared to extended PLND. Luiting et al. retrospectively reviewed the literature to evaluate the role of ^68^Ga-PSMA PET in detecting lymph node metastases in men with prostate cancer [23]. They found that ^68^Ga-PSMA PET is more accurate than other imaging modalities in detecting lymph node metastases and can guide the extent of dissection. It is worth noting that the authors also found that the accuracy of ^68^Ga-PSMA PET was influenced by the number of nodes removed during extended PLND. This suggests that the more nodes that are removed, the more accurate ^68^Ga-PSMA PET will be in detecting lymph node metastases. Luiting et al.’s results suggest that ^68^Ga-PSMA PET may be a more accurate way to stage prostate cancer and guide treatment decisions [23].

PLND is the most accurate method for detecting whether prostate cancer has spread to the pelvic lymph nodes. In men who have localized prostate cancer (cancer that has not spread outside of the prostate), PLND may be performed as part of a radical prostatectomy (surgery to remove the prostate). In men who have already had a radical prostatectomy, PLND may be performed as part of a salvage prostatectomy (surgery to remove the prostate after radiation therapy has failed to control cancer). A study by Van Huele et al. found that minimally invasive surgery is a safe and effective approach to performing a PLND because it is associated with less blood loss, less need for transfusions, shorter hospital stays, and fewer complications than open surgery [24]. Lymph node yield was significantly higher in the RA group than in the laparoscopic group and the open group [24]. The authors also found that the risk of lymph node metastasis (the spread of cancer to the lymph nodes) was significantly lower in the RA group than in the laparoscopic and open groups) [24]. The number of positive lymph nodes was an important predictor of worse survival on multivariable analysis, demonstrating the need for accurate node staging. Thus, the authors concluded that a minimally invasive, robot-assisted PLND is a safe and effective approach to staging prostate cancer.

Lymph node staging of prostate carcinoma is important because the stage is the most important prognostic factor for patients with this disease. The number of positive lymph nodes is an important predictor of recurrence and survival. A study by Sprute et al. proved that the tracer ^18^F-PSMA-1007 has high sensitivity and specificity in detecting lymph node metastases in prostate cancer patients [25]. They showed that PSMA-1007 is a promising new tracer for staging lymph node metastases in prostate cancer patients. This is important because lymph node metastases are the most common cause of death from prostate cancer. According to Sprute et al., in a regional analysis, the sensitivity of ^68^Ga-PSMA-11 PET/MRI was 72-100%, whereas specificity was 96-100%. This tracer can be used to accurately stage lymph node metastases in prostate cancer patients [25].

This analysis shows a plausible positive impact of using this treatment on patients with lymph node metastasis. This is important because it suggests that ^68^Ga-PSMA PET may be a more accurate way to stage prostate cancer and guide treatment decisions. Because lymph node metastases are the most common cause of death from prostate cancer, this treatment could potentially save lives. Furthermore, the tracer ^18^F-PSMA-1007 has high sensitivity and specificity for prostate cancer, so this could be a more accurate way to diagnose the disease. This is important because it could lead to earlier treatment and potentially better patient outcomes. Furthermore, the minimally invasive, robot-assisted PLND is a safe and effective approach to staging prostate cancer, which could be used as a less invasive option for patients. This is important because it can lead to less blood loss, shorter hospital stays, and fewer complications.

Plausible Positive Impact: Any PSA Decline After a Few Cycles

Satapathy et al. reported that the best PSA was achieved in 50% of the studied patients, with 10 out of 20 treated with ^177^Lu-PSMA-617 compared to 40%, eight out of 20 patients treated with docetaxel [26]. Patients were treated with ^177^Lu-PSMA-167 every eight weeks for one to four cycles while the docetaxel group was treated every three weeks for up to 10 cycles, with adverse events occurring less frequently in patients treated with ^177^Lu-PSMA-167. With ^177^Lu-PSMA-617 shown to be safe and non-inferior in treatment to docetaxel, ^177^LU-PSMA-617 could be used for treatment earlier rather than being reserved for advanced end-stage metastatic castration-resistant prostate cancer [26].

In the study performed by Barber et al., patients were classified as taxane chemotherapy pretreated (T-pretreated) or naïve (T-naïve), not previously treated with taxane chemotherapy [26]. Patients were treated with ^177^Lu-PSMA, 83 patients were T-pretreated and 84 were T-naïve. PSA was evaluated in 132 patients, 62 T-pretreated and 70 T-naïve. A decline in PSA was seen in 40% of T-pretreated patients and 57% of T-naïve patients, with the average number of cycles treated with ^177^Lu-PSMA being two to four cycles and one to three cycles, respectively [27]. The overall survival was found to be increased in each group of patients treated with ^177^Lu-PSMA, with increased overall survival in T-naïve patients with only minimal grade 3 or 4 adverse events present in the follow-ups of either group [27]. It is suggested that ^177^Lu-PSMA-617 is a valid treatment option for not only patients with metastatic castration-resistant prostate cancer but also for patients with less advanced forms of prostate cancer.

Ahmadzadehfar et al. evaluated the hematotoxicity in patients pre-exposed to ^223^Ra-dichloride who received three cycles of ^177^Lu-PSMA-617 compared to patients without previous exposure to ^223^Ra-dichloride [1]. Patients were treated with three cycles of ^177^Lu-PSMA-617 with eight weeks between cycles, with each patient having at least two follow-ups after the third cycle. A PSA decline of greater than 50% two months after the third cycle of ^177^Lu-PSMA in 53.1% was found in the patients included in this study, with more than 60% of the included patients not showing any hematotoxicity [1]. A higher percentage of participants of the group with no previous ^223^Ra exposure showed greater than 50% PSA decline compared to participants with previous exposure to ^223^Ra. It was concluded that ^177^Lu-PSMA-617 radioligand therapy is a safe option for patients with previous exposure to radiation therapy, with no increase in radiation-induced hematotoxicity as well as patients with no previous exposure to ^223^Ra-dichloride [2].

In the study conducted by Gadot et al., ^177^Lu-PSMA-617 was given as a last line, and the study was self-funded so more than one treatment was not automatically offered [11]. PSA levels were recorded at nadir, with PSA nadir being defined as the minimal PSA throughout the follow-up period. Patients were to return for safety assessment within four weeks after the first treatment. Overall, 58% of patients showed a decline at first PSA, with 35% showing greater than 50% PSA decline. A PSA decline of 50% or above was significantly associated with overall survival, with average survival increasing from 3.5 months for patients without the PSA decline to 11 months for patients with 50% or greater PSA decline [11]. This study shows that ^177^Lu-PSMA-617 is beneficial to patients with metastatic castration-resistant prostate cancer and that PSA decline is associated with overall survival [9].

The study by Scher et al. aimed to evaluate and revise previous castration-resistant prostate cancer clinical trial recommendations from previous prostate cancer clinical trial working groups. The primary endpoint was the best PSA response rate which was defined according to the Prostate Cancer Clinical Trials Working Group-3 (PCWG3) as the proportion of patients achieving a ≥50% decline in PSA from baseline [28]. PCWG3 suggests the outcomes for PSA should be interpreted within the context of the drug’s mechanism of action, along with the timing of a potential favorable or unfavorable effect of protein-specific antigen. They also suggest that response to treatment whether sensitive or resistant should be categorized based on post-therapy PSA. An increase in PSA is a sign of tumor growth followed by disease worsening and increased clinical symptoms [28]. PCWG3 has advised recording whether tumor progression was indicated by PSA alone, bone or node location, or bone plus node location, along with the proportion of patients who progress because this is prognostic [28]. PCWG3 is against activity-estimating endpoints in early-phase trials (such as declines in PSA) where the objective is to demonstrate sufficient antitumor activity to justify further study. It is recommended to assess PSA levels by cycle or every three or four weeks and to continue therapy if an isolated PSA rise occurs after an initial decline until radiographic or clinical symptoms worsen [28].

Plausible Positive Impact: More Than 50% PSA Decline After a Few Cycles

Docetaxel is a medication that contains metal gallium (^68^Ga) in the treatment of mCRPC. First, the drug binds to the PSMA, which is highly expressed in prostate cancers. The drug is incorporated in prostate cancer cells, then the binding Ga-PSMA is excreted through urine. PET-CT is used to check the size and progression of the tumor. The PSA monitors the treatment and how effective the drug is. Sixteen patients with follow-up docetaxel therapy and PET-CT scan were included in the study (between 52 to 82 years of age with a mean age of 69 years). PET-CT was done and PSA levels were measured before and after the administration of docetaxel and after at least three cycles of chemotherapy. The images were then compared for the effectiveness of the drug or cancer progression [29].

A combination of docetaxel and prednisone was the first treatment to prolong survival in men with mCRPC. Docetaxel inhibits microtubular depolymerization and antiproliferation. The incorporation of prednisone potentially augments the efficacy of docetaxel in patients within mCRPC. It is limited to patients who have not previously received corticosteroids. In an experiment in China, 16 patients participated in docetaxel plus prednisone chemotherapy between August 2011 and May 2019 with a mean age of 82 years (range = 80-87 years). Ten (62.50%) patients had a good PSA response of ≥50% decline. All patients received a median of four and a half cycles of three-week docetaxel regimens and 5 mg prednisone twice per day. Hematologic toxicity was detected in six patients with neutropenia, and one patient had agranulocytosis. Other adverse reactions such as fever, debilitation, and alopecia were also observed [25].

Another experiment used inecalcitol as a clinical phase 1 trial [30]. Its goal was to test the safety of unhealthy volunteers. The efficacy of the drugs was evaluated in groups of three to six patients receiving the medicine in 21 days. Inecalcitol is a novel vitamin D receptor agonist with a higher antiproliferative effect. A vitamin D analog binds to the vitamin D receptor and promotes the formation of a heterodimer complex with the retinoid x receptor which results in target gene transcription and antiproliferation effect by reducing cellular invasiveness and inducing apoptosis. They had, respectively, 85% and 76% of patients with a ≥30% PSA decline within three months and a ≥50% PSA decline at any time. They treated 54 patients with inecalcitol, and the maximum tolerated dose was 4,000 μg daily. Safety and efficacy were evaluated in groups of three to six patients receiving inecalcitol during a 21-day cycle in combination with docetaxel (75 mg/m^2^ every three weeks) and oral prednisone (5 mg twice a day) for up to six cycles. The primary endpoint was dose-limiting toxicity defined as grade 3 hypercalcemia within the first cycle. The efficacy endpoint was a ≥30% PSA decline within three months.

Plausible Positive Impact: Better Performance Status

Small-molecule signal transduction inhibitors are the standard therapy in renal cell carcinoma and are currently being evaluated for the treatment of prostate cancer [31]. The inhibitors are aimed at the pathway involved in prostate cancer progression. They provide selective therapies that are aimed at the molecules that are critical to cellular signaling in prostate cancer. The PI3K/Akt/mTOR pathway plays a prominent role in prostate cancer [28]. In 50% of prostate cancer, the PI3K/Akt/mTOR is seen to be upregulated due to the loss of PTEN. The molecular changes seen in this pathway determine whether the cancer is benign or malignant. There are multiple inhibitors of the pathway that have been developed; one of them is the mTOR inhibition in combination with traditional chemotherapies, for example, bevacizumab, gefitinib, and docetaxel. This has been shown to decrease the rate of prostate cancer [31].

In prostate cancer, bone is the most common site of metastases in patients where cancer has progressed beyond organ confinement [32]. Bone metastasis is incurable and leads to mortality. Bone provides environments that are conducive for tumor cells to be targeted for therapeutic interventions. Many molecules and pathways have been identified as new potential therapeutic targets for bone metastases caused by metastatic castration-resistant prostate cancer [32]. The therapies covered are agents that target and inhibit bone resorption, which includes agents that stimulate bone formation as well as agents that target bone matrix. The bone is targeted by presenting a bone-targeting polymer vesicle with excellent single-photon emission computed tomography imaging. Tomography allows the efficient delivery of antitumor drugs into the bone [30]. Bone-targeting polymer vesicles provide a platform that provides real-time diagnosis and therapy of malignant bone tumors. Bone matrix therapies have been developed those target bone resorption agents that stimulate bone formation and target bone matrix. These targeted therapies increase the survival rate and quality of life.

Prostate cancer has mainly been treated by ADT. Many patients respond to ADT initially but relapse and develop resistance to therapy as the disease progresses [17]. A better understanding of the mechanism that provides resistance has led to the development of androgen receptor targeting agents. Some of these target agents are abiraterone, acetate, and enzalutamide. The evolution of ADTs is useful for the treatment of advanced-stage prostate cancer. The aim is to target androgen receptors for the treatment of advanced-stage prostate cancer. This is because extragonadal androgen contributes to the progression of castration-resistant prostate cancer. Abiraterone acetate is an inhibitor of androgen biosynthesis and prolongs overall survival among patients with metastatic castration-resistant cancer. An experiment showed that the survival rate was longer for patients using abiraterone than for patients who got the placebo [17]. The inhibition of androgen biosynthesis by abiraterone has prolonged overall survival among patients with metastatic castration-resistant prostate cancer.

Lipogenic alterations that occur in prostate cancer are overexpression of the enzyme fatty acid synthases (FASN) and deregulation of the 5-AMP-activated protein kinase (AMPK) [4]. Targeting the metabolic pathways that may have been altered during prostate tumorigenesis and progression has been in prostate cancer prevention and treatment. Alterations in cellular metabolism are strongly linked with oncogene activation and silencing of tumor suppressor genes. These alterations are being targeted for the diagnosis and treatment of prostate cancer. Lipogenic enzymes FASN and AMPK are targeted in lipogenic pathway-inhibiting therapies [4].

Targeted therapy in cancer involves the use of drugs to target the biological pathways of tumor cells by inhibiting the pathway. The use of investigation at the molecular level of cancer cells has helped in finding out why many anticancer drugs become resistant. Drug resistance in patient therapy impacts the survival and prognosis of patients with cancer. Molecular-level cancer therapy is essential for anticancer drug improvement. Large amounts of fatty acids and cholesterol are synthesized by cancer cells [4]. Lipogenic alterations that commonly occur in prostate cancer are overexpression of the enzyme FASN and deregulation of the AMPK [4]. Fatty acid synthase is a key metabolic enzyme that plays a role in energy homeostasis. It converts excess carbon intake into fatty acids for storage. AMPK functions as a central metabolic switch that governs glucose and lipid metabolism [4]. Recent interest has focused on the potential of targeting metabolic pathways that may be altered during prostate tumorigenesis and progression are therapeutic for cancer. Drugs that directly or indirectly induce AMPK activation have potential benefits in prostate cancer prevention and treatment.

Plausible Negative Impact: Visceral Metastasis

In a study from Theranostics [33], 109 patients who were confirmed to have mCRPC were treated with a median of three cycles of Lu-PSMA-617. The decline of PSA was determined by observing the initial PSA levels from the first cycle and contrasting that with the best PSA response from the entire observation period. All patients had been treated previously with at least one line of chemotherapy and at least one next-generation anti-hormonal therapy such as abiraterone or enzalutamide. The results showed that 54 (49.5%) patients had died during the observation period. Of that percentage, 55% showed an initial PSA decline, while 25% had shown a PSA decline of more than 50%. It was observed that there was a clear difference in the overall survival of patients treated with cabazitaxel versus those who were cabazitaxel-naive. Those who had been treated with cabazitaxel were observed to have more instances of developing visceral metastasis, which was associated in the study to have a correlation with a lower overall survival rate.

Discussion

Targeted therapies are an important and effective treatment option for advanced metastatic prostate cancer. In a study published by Yadav et al., molecular-targeted therapy using radioligand ^177^Lu-PSMA-67 was found to be effective in treating patients with metastatic disease [33]. This therapy has minimal side effects and can be used in combination with other treatments. In this study, the long-term follow-up and outcomes of patients treated with ^177^Lu-PSMA-617 theranostics revealed that this therapy is safe and effective. This therapy should be considered a treatment option for patients with mCRPC.

In a study by Kessel et al., ^177^Lu-PSMA (a radioligand therapy) was used to treat advanced metastatic prostate cancer [32]. The study found that ^177^Lu-PSMA was an effective treatment for advanced metastatic prostate cancer. The study also found that the side effects of ^177^Lu-PSMA were manageable and that the treatment was well tolerated by patients. Radioligand therapy is a type of cancer treatment that uses radioactive substances to target and kill cancer cells. PSMA is a protein that is found on the surface of prostate cancer cells. ^177^Lu-PSMA binds to PSMA and delivers a radioactive payload to the cancer cell, killing it. Radioligand therapy is a type of targeted therapy, which means that it targets specific proteins that are found on the surface of cancer cells. Targeted therapies are often more effective than traditional chemotherapy because they specifically target cancer cells, while traditional chemotherapy can damage healthy cells as well as cancer cells.

Limitations

There were some notable limitations of our review. Because this was a narrative review, we were unable to collect independent data as part of our own clinical review due to a lack of resources. There was also no singular limitation on the administered cycles and as such we reported as many as five cycles of ^177^Lu-PSMA-617 in some clinical experiments. Also, there was an inconsistent number of patients who had a varying number of previous therapies administered which could have altered the results significantly. Going forward, there needs to be a set amount of previous therapies and age ranges reported in the review. Furthermore, the early administration of ^177^Lu-PSMA-617 was rarely discussed and could have been a major prognostic factor that greatly impacted overall survival in mCRPC patients. In future reviews, these parameters will be explored further to provide a more thorough scope of the benefits of ^177^Lu-PSMA-617.

## Conclusions

Prostate cancer is the fourth leading cause of cancer in the United States and the most prevalent cancer among men specifically. Patients with advanced cancer who become unresponsive to ADTs are referred to as having mCRPC which has shown a positive treatment response with ^177^Lu-PSMA-617. In our narrative review, we found that the first cycle of ^177^Lu-PSMA therapy was the most effective parameter that proved a higher likelihood of survival in mCRPC patients. Moreover, lymph node metastasis proved not to be detrimental as a prognostic factor in determining the overall survival of patients as many studies reported positive results with a greater amount of metastasis. On the other hand, the Gleason score was found to be significant in determining response prediction and overall survival in patients. Multiple studies showed a decrease in PSA levels of more than or greater than 30% after one or more cycles of ^177^Lu-PSMA-617. Therefore, this radioligand therapy is a viable form of treatment for those with both non-metastatic and advanced metastatic prostate cancer.
